# MicroRNA-21a-5p Functions on the Regulation of Melanogenesis by Targeting Sox5 in Mouse Skin Melanocytes

**DOI:** 10.3390/ijms17070959

**Published:** 2016-06-24

**Authors:** Pengchao Wang, Yuanyuan Zhao, Ruiwen Fan, Tianzhi Chen, Changsheng Dong

**Affiliations:** 1 College of Animal Science and Technology, Shanxi Agricultural University, Taigu 030801, Shanxi, China; wangsh0402@163.com (P.W.); ruiwenfan@163.com (R.F.); chentianzhi15@163.com (T.C.); 2 Wujiang River Institute of Agriculture & Forestry Economics, Tongren University, Tongren 554300, Guizhou, China; 84840293@163.com

**Keywords:** melanocytes, Sox5, miR-21a-5p, transcriptional regulation

## Abstract

MicroRNAs (miRNAs) play an important role in regulating almost all biological processes. miRNAs bind to the 3′ untranslated region (UTR) of mRNAs by sequence matching. In a previous study, we demonstrated that miR-21 was differently expressed in alpaca skin with different hair color. However, the molecular and cellular mechanisms for miR-21 to regulate the coat color are not yet completely understood. In this study, we transfected miR-21a-5p into mouse melanocytes and demonstrated its function on melanogenesis of miR-21a-5p by targeting Sox5, which inhibits melanogenesis in mouse melanocytes. The results suggested that miR-21a-5p targeted Sox5 gene based on the binding site in 3′ UTR of Sox5 and overexpression of miR-21a-5p significantly down-regulated Sox5 mRNA and protein expression. Meanwhile, mRNA and protein expression of microphthalmia transcription factor (MITF) and Tyrosinase (TYR) were up-regulated, which subsequently make the melanin production in melanocytes increased. The results suggest that miR-21a-5p regulates melanogenesis via MITF by targeting Sox5.

## 1. Introduction

The synthesis and distribution of melanins determine pigmentation. Melanin has two main types: brown/black eumelanin and red/yellow pheomelanin [1]. Melanin is synthesized in the melanosomes of melanocytes and transferred to the neighboring keratinocytes, where the accumulation of melanins generates pigmented skin or hairs [2,3]. Melanin granules are transferred to cortical and medullary keratinocytes and form pigmented hair shafts [4]. Melanin is a protective pigment and absorbs ultraviolet (UV) irradiation in skin function [5]. Many members of the Sox protein family are important developmental regulators and extensively expressed in the vertebrate embryo [6,7]. Sox5 is one of the SoxD transcription factors and also a key cell fate modulator [8]. Moreover, Sox5 is the major SoxD protein in melanoblasts, and it occurs in the neural crest cells of mouse. It is implicated in regulating the life cycle of melanocyte and participates as a transcription factor that affects key melanocytic genes in both regulatory and modulatory manners [9,10]. Microphthalmia transcription factor (MITF) is a major regulator of melanocyte development, and expresses in the process that distinguishes a melanoblast from a migrating neural crest cell [11]. However, melanin pigmentation in mammalian skin is also regulated by many factors, including UV radiation, hormones, and genes [12]. MITF is one of these factors. As hormone-like regulators, l-tyrosine and l-DOPA play an important role in melanocyte functions [13].

miRNAs play an significant role in the regulation of gene by targeting genes with sequences complemented to the mature miRNA sequence and then regulating targeted genes expression by various mechanisms [14]. A number of studies about the function and expression of miRNAs in the skin of mammalian species, including mouse, goat, sheep and alpaca, have been reported [3,15,16]. Studies about miRNAs functions on melanogenesis are limited, though many miRNAs have been shown to play significant role in melanoma [17,18]. Some miRNAs can change coat color. As an example, the mouse skin color changes from black to brown when overexpression of miR-137 in mouse [19].

The expression of miR-21 has a significant difference between in white alpaca skin and brown skin [20]. In our work, we investigated the functional role of miR-21a-5p in promoting melanogenesis in mouse melanocytes.

## 2. Results and Discussion

### 2.1. miR-21a-5p Targets the 3′ Untranslated Region (UTR) of Sox5 mRNA

**Figure 1:**
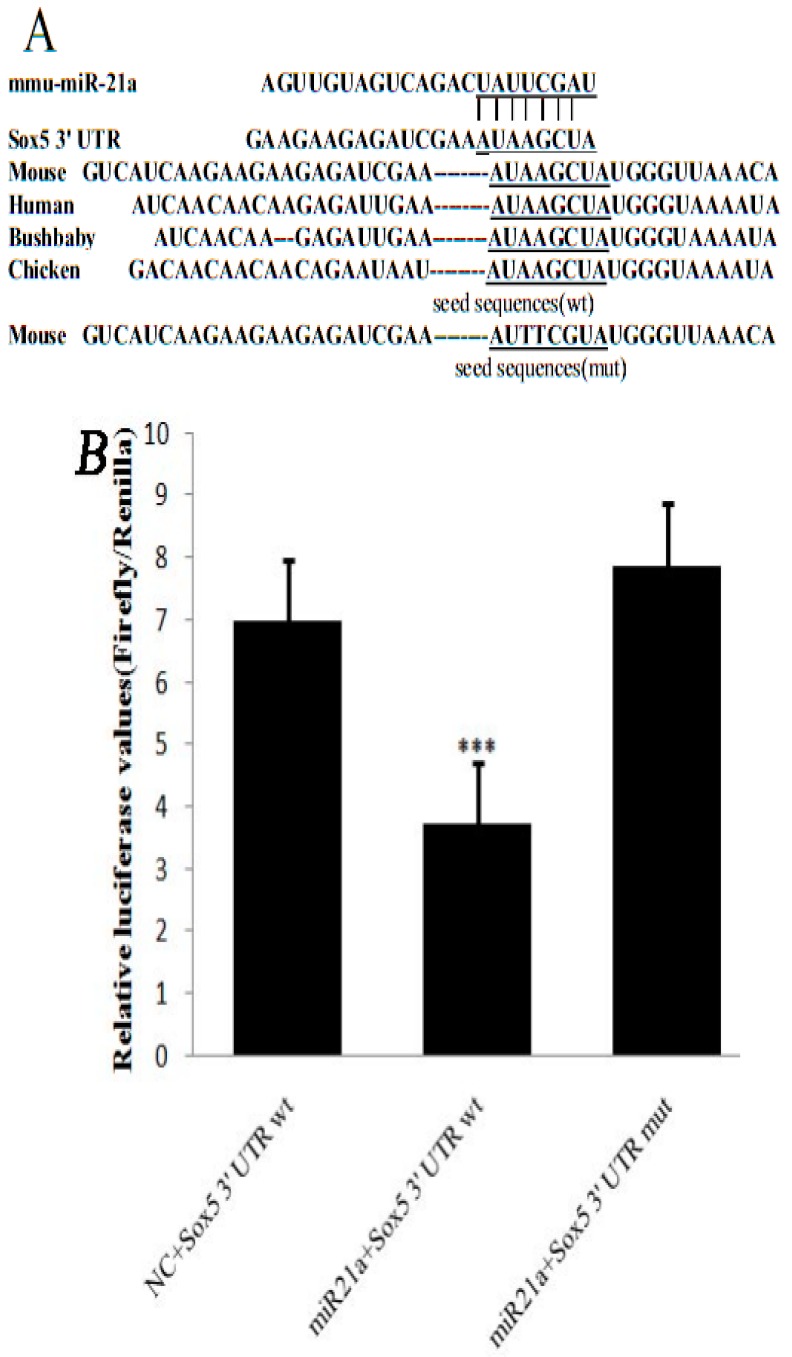
Sox5 is a target of miR-21a-5p: (**A**) the sequences of Sox5 3′ untranslated region (UTR) around the miR-21a-5p binding site from its homology across species; and (**B**) relative luciferase activity transfected with reporter constructs containing the wild-type (wt) or mutated (mut) seed sequences of pmiRGL0-Sox5 together with pcDNA6.2™-GW/EmGFP-miR-21a-5p (miR-21a) or the negative control plasmid (NC). The underline sequence are binding site. *** *p* < 0.001.

The target genes of miR-21a-5p were predicted using TargetScan (http://www.targetscan.org/) and miRanda (http://www.microRNA.org/). Sox5 involved in melanogenesis among the common target genes (Figure 1A). Through the forecast miRNA integration point to confirm the characteristic of miR-21a-5p convention of Sox5, not only the wild type Sox5 3′ UTR (pmiRGL0-Sox5-wt-3′ UTR) but also the mutant Sox5 3′ UTR (pmiRGL0-Sox5-mut-3′ UTR) was contained when luciferase reporter assays were done using luciferase reporter constructs. The constructs were co-transfected into HEK293T cells with the pcDNA6.2™-GW/EmGFP-miR-21a-5p expression plasmid or negative control plasmid (NC). The results showed that the luciferase activity in cells co-transfected by pmiRGL0-Sox5-wt-3′ UTR and pcDNA6.2™-GW/EmGFP-miR-21a-5p was decreased by 46% compared with that in cells of NC. No difference in luciferase activity was found between the cells co-transfected with pcDNA6.2™-GW/EmGFP-miR-21a-5p and those with pmiRGL0-Sox5-mut-3′ UTR (Figure 1B). The results indicated that miR-21a-5p could bind and regulate the 3′ UTR of Sox5.

### 2.2. Effect of miR-21a-5p Overexpression on miR-21a-5p and Sox5 Expression

**Figure 2:**
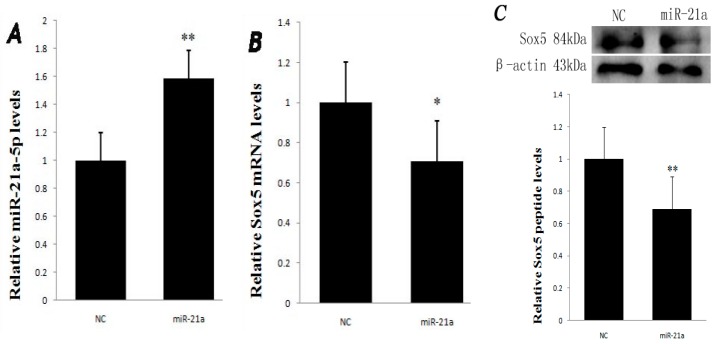
Expression levels of miR-21a-5p and Sox5 mRNA in NC and miR-21a-5p transfected groups: (**A**) the miR-21a-5p expression level was significantly higher in the miR-21a group than in the NC group (miR-21a: 1.59, NC: 1); (**B**) many significant differences were found in the Sox5 mRNA expression between the NC and miR-21a groups (miR-21a: 0.71, NC: 1); and (**C**) the Sox5 protein amount in melanocytes transfected with miR-21a as well as the negative control. Data are mean ± SD. * *p* < 0.05, ** *p* < 0.01.

The expression of mRNA and protein for Sox5 was determined in melanocytes transfected by the miR-21a-5p overexpression vector. As shown in Figure 2A, the result showed that the expression of miR-21a-5p was significantly higher in the miR-21a-5p transfected group than that in the negative control (NC) group with a significant difference (*p* < 0.01). However, the overexpression of miR-21a-5p resulted in the expression of Sox5 mRNA decreased (*p* < 0.05, Figure 2B). Meanwhile, the level of Sox5 protein was decreased in the miR-21a-5p transfected group compared with that in the NC group (*p* < 0.01, Figure 2C). The result intimates that the overexpression of miR-21a-5p inhibits the Sox5 expression at the protein level. The results reveals that miR-21a-5p might regulate Sox5 protein translation by affecting Sox5 expression, consistent with the rules of miRNA actions on the target genes [21,22].

### 2.3. Effect of miR-21a-5p Overexpression on Microphthalmia Transcription Factor (MITF) and Tyrosinase (TYR) Expression

**Figure 3:**
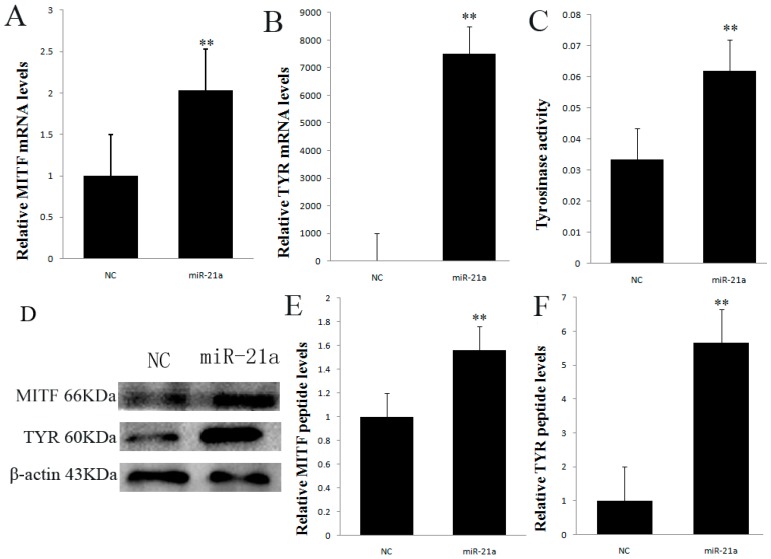
(**A**) The mRNA expression levels of microphthalmia transcription factor (MITF); (**B**) The mRNA expression levels of Tyrosinase (TYR); (**C**) Tyrosinase activity in melanocytes transfected with miR-21a plasmid and their negative control; (**D**) Western blot analysis of MITF and TYR protein expression; (**E**) Expression levels of MITF protein; (**F**) Expression levels of TYR protein. Data are mean ± SD. ** *p* < 0.01.

The expression of mRNA and protein for MITF and Tyrosinase (TYR) was examined in melanocytes transfected by the miR-21a-5p overexpression construct. As shown in Figure 3A,B, quantitative real-time PCR (Polymerase Chain Reaction ) analysis result showed that the expression of MITF and TYR was significantly higher in the miR-21a-5p transfected group than in the NC group with a significant difference (*p* < 0.01). The expressions of MITF and TYR protein in the miR-21a or NC group were determined by Western blotting, respectively (Figure 3D). Meanwhile, the level of MITF and TYR protein were increased in the miR-21a group compared with that in the NC group (*p* < 0.01, Figure 3E,F). The tyrosinase activity was examined in melanocytes transfected by the miR-21a-5p overexpression construct. As shown in Figure 3C, the data suggested that the tyrosinase activity in the miR-21a group was significantly higher than that in the NC group.

### 2.4. Effect of miR-21a-5p Overexpression on the Cell Pellets

**Figure 4:**
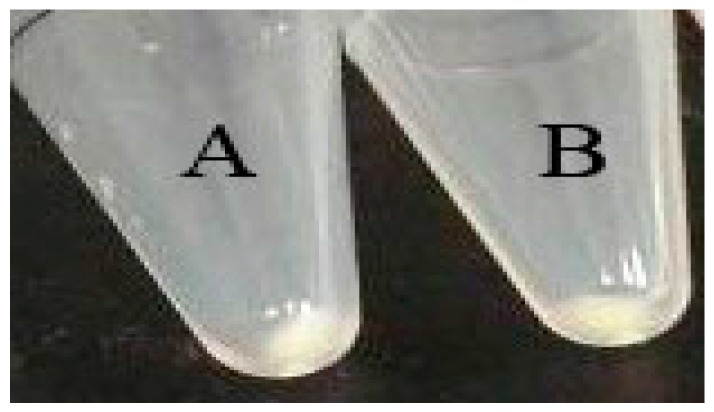
Effect of miR-21a on cell pellets. Melanocytes transfected with: miR-21a plasmid (**A**); and NC (**B**).

Melanocytes were transfected with miR-21a-5p and NC to determine the effect of miR-21-5p on the pellets of melanin. Melanocytes were collected by centrifugation. The results showed that there was no obvious effect on melanin pigmentation in the miR-21a transfected group compared with that in the NC group (Figure 4).

This paper is the first time to examine how miR-21a-5p inhibits Sox5 expression and promotes melanogenesis by regulating MITF expression in mouse melanocytes. The results are consistent with a previous study, which found that the transcriptional activation of MITF promoters could be inhibited by the binding of Sox5 to the regulatory regions of MITF [9]. Mutations of Sox5 affecting the MITF pathway lead to pigmentary and auditory defects [1]. Therefore, the functional role of MITF in the control of genes important for coat color/pigmentation was well established. A significantly enhanced expression of MITF (>2 fold) was noted in mouse skin melanocytes in the miR-21-5p group vs. the negative control group (Figure 3A). According to some studies, Sox5 is a molecular switch which determines the xanthophore versus leucophore fate and the pigment cell fate choice from the shared progenitorand neural crest cells (NCCs), respectively [23]. Sox5 also ensures the proper development of specific neuronal cell types by controlling the timing of critical cell fate and differentiation decisions [24,25]. Sox5 can control cell cycle progression in neural progenitors by interfering with the WNT–β-catenin pathway [26]. The WNT–β-catenin pathway is also present in melanocyte regulation. Sox5 potentially affects the WNT–β-catenin pathway in the regulation of melanin. However, overexpression of Sox5 on chick embryo promotes the generation of the neural crest [27]. In all vertebrates, melanocytes life cycle starts with the emergence of the NCCs from the dorsal neural tube [28]. The NCCs destined to become melanocytes immediately upregulate melanocyte-specific genes beginning with MITF [29,30,31,32]. Thus, the above evidence suggests that a link exists between Sox5 and MITF as Sox5 binds to the regulatory regions of MITF and inhibits MITF activity [9]. miR-21a-5p can repress Sox5 to promote MITF and melanogenesis in mouse melanocytes.

Many miRNAs can affect melanin. In the process of melanogenesis, many miRNAs play a crucial role in governing the regulation of melanin. For example, miR-340 and miR-145 have been shown to participated in the pigmentation [33,34]. miR-434-5p was discovered to inhibit TYR to turn mouse skin white [35]. Moreover, miR-21 was widely expressed in other tissues [36]. For example, miR-21 prevented the inhibitory effects of BMP4 on cell proliferation and migration in primary keratinocytes and HaCaT cells [37]. In addition, the up-regulation of miR-21 resulted in decreased apoptosis and increased proliferation in melanocytes [38]. miR-21 has many target genes such as PDCD4 (programmedcelldeathprotein4) [39], PTEN (phosphate and tension homology deleted on chromsome ten) [40], P53 (tumor suppressor P53) [41], TIMP3 (tissue inhibitor of metalloproteinase 3) [42], etc. Chen et al. [43] found that Sox5 was one of many targets of miR-21. Our experiment found that the gene mutation of Sox5 was the target gene of miR-21a-5p. The overexpression of miR-21a-5p in mouse melanocytes downregulated the expression of Sox5 both at the transcriptional and translational levels (Figure 2B,C). Therefore, miR-21a-5p may play a significant role in melanogenesis in mouse melanocytes by regulating Sox5, which is a potential hair color gene. In present study, we provided evidence supporting the role of miR-21a-5p in promoting melanogenesis (Figure 3). The tyrosinase activity and other melanogenesis related proteins can be stimulated without an increase in melanin pigmentation in certain models [44]. This may be related to the fact that melanogenesis can be differentially regulated at the levels of gene expression, protein expression, their processing, melanosome formation translocation of tyrosinase to the melanosomes and enzyme activation [12,45,46,47,48]. Therefore, it was interesting that the stimulation of melanogeneic genes caused by miR-21a-5p didn’t result in the stimulation of melanin. Although many studies have confirmed that miRNAs are involved in regulating melanogenesis, few studies have focused on the formation and transport of melanosomes in miRNA function. Consequently, studies should focus on the miRNA function in the future.

## 3. Material and Methods

### 3.1. Sample Material Selection

This study and melanocytes of mouse source were permitted by Ethics Committee of Shanxi Agricultural University, Taigu, 030801, China.

### 3.2. Plasmids

Mouse miR-21a-5p expression plasmid was constituted by inserting an oligonucleotide conforming to the sequence of the mmu-miR-21a-5p into a mammalian expression vector, pcDNA6.2-GW/EmGFPmiR (Invitrogen, Carlsbad, CA, USA).

Fragment (249 bp) of the mouse Sox5 3′ UTR was PCR amplified by Sox5 primers and then inserted into pmiRGL0 dual-luciferase miRNA target vector (Promega, Fitchburg, WI, USA) with Sal I and Xho I restriction sites to form the pmiRGL0-Sox5-wt-3′ UTR construct. The pmiRGL0-Sox5-mut-3′ UTR construct was created by Sox5 specially designated primers using the KOD-Plus-Mutagenesis Kit (TOYOBO CO., OSAKA, Japan) (Table 1).

### 3.3. Cell Culture

Melanocytes were established in Alpaca Biological Engineering Laboratory, Shanxi Agricultural University, Taigu, China. Melanocytes were cultured in MelM (ScienCell, Carlsbad, CA, USA). HEK 293T cells were cultured in DMEM (Gibco, New York, NY, USA) complemented fetal bovine serum (FBS) (10%).

Melanocytes were transfected with miR-21a-5p plasmid or the negative control plasmid using X-tremeGENE HP DNA Transfection Reagent (Roche Diagnostics GmbH, Mannheim, Germany). Two days after the cells were collected to extract total RNA and protein.

### 3.4. Luciferase Reporter Assay

Two days after transfection of HEK293T cells with 0.15 µg of pmiRGL0-Sox5-wt-3′ UTR or pmiRGL0-Sox5-mut-3′ UTR together with 0.25 µg of miR-21a-5p or negative control, cells were dissolved in 1× Passive Lysis Buffer. Then, the luciferase activity was determined using GLOMAXTM 96 Microplate Luminometer (Promega, Madison, WI, USA), which was decided by the Firefly luciferase values normalized to the Renilla luciferase values.

### 3.5. Quantitative Real-Time PCR Analysis

Following the total RNA of melanocytes being extracted using TRIzol reagent (Invitrogen, Carlsbad, CA, USA), cDNA was synthesized for the quantitative real-time PCR analysis of miR-21a-5p using cDNA synthesis kit (TAKARA, Dalian, China) and a miR-21 stem loop primer (Table 1). The expression of miR-21a-5p was performed by miR-21 sequence-specific forward primer (Table 1) using SYBR^®^ PrimeScript™II RT-PCR kit (TAKARA, Dalian, China), on the StepOnePlus Real-Time PCR system for triplicate (Life Technologies, Grand Island, NE, USA). The quantification of miR-21a-5p abundance was determined using the 2^−ΔΔ*C*t^ method normalized to U6. The analysis of mRNA abundance was performed for Sox5, TYR and MITF using gene specific primers normalized to 18S rRNA.

### 3.6. Western Blot Analysis

The cell lysates were separated on 10% gels by SDS-PAGE electrophoresis and then transferred to nitrocellulose filter membranes (Millipore, New York, NY, USA). After the membranes were blocked in 5% evaporated skimmed milk (Boster, Wuhan, China) at 25 °C for 1 h and washed five times using Tris-buffered saline-Tween (TBST) for 8 min each time, it was incubated with anti-Sox5 primary antibody at 1:800 dilution (from rabbit, Abcam, Cambridge, MA, USA), anti-MITF primary antibody at 1:500 dilution (from rabbit, Abcam, Cambridge, MA, USA), anti-TYR primary antibody at 1:1000 dilution (from rabbit, Abcam, Cambridge, MA, USA) and anti-β-actin primary antibody at 1:2000 dilution (from rabbit, Boster, Wuhan, China) overnight at 4 °C, respectively. Following washing four times in TBST for 8 min each, the membranes were incubated with horseradish peroxidase (HRP)-conjugated goat anti-rabbit-IgG at 1:2000 (Boster, Wuhan, China) at 37 °C for 1.5 h. Subsequently, a super ECL chemiluminescence plus (Boster, Wuhan, China) was used for visualization after the membranes were washed. The relative intensities of the above proteins were analyzed by Image Lab software (Bio-Rad Laboratories, Philadelphia, PA, USA).

### 3.7. Determination of Tyrosinase Activity in Melanocytes by DOPA Oxidation Reaction

Transfected cells were added 90 µL concentration 1% TritonX-100 solution in each hole and placed in −80 °C refrigerator frozen stored for 30 min and made cell lysis completely. Then, 10 µL 0.1% l-DOPA solution was added in each hole and incubated for 2 h in an incubator. The absorbance A value was measured at 490 nm using a Multiscan Spectrum microplate reader (Thermo, Waltham, MA, USA).

### 3.8. Observation of Cell Pellets

Melanocytes were transfected with miR-21a-5p plasmid or the negative control plasmid. Two days after, the cells from each condition were centrifuged into pellets for the purpose of photography.

### 3.9. Statistical Analysis

The differences of mRNA and protein in Sox5, TYR and MITF in miR-21a transfected group and NC group were measured by SPSS 17.0 software (IBM, Chicago, IL, USA).

## 4. Conclusions

**Figure 5:**
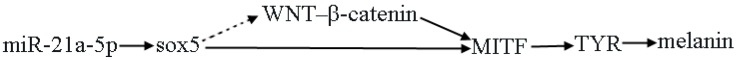
Potential pathway of miR-21a-5p in regulating melanogenesis in melanocytes of mouse.

In summary, our data support the important functional role of miR-21a-5p by targeting Sox5 in mouse melanocytes. Sox5 binds to the regulatory regions of MITF, which regulates melanogenesis through the MITF pathway. The proposed pathway for the miR-21a-5p regulation of melanogenesis in mouse melanocytes is portrayed in Figure 5, which also showed the potential mechanism of melanogenesis through the WNT–β-catenin pathway. Thus, it is one of the candidate genes for producing natural wool fiber in fiber-producing species through the genetically modified method.

## Figures and Tables

**Table 1:** Primers used in this study. The underline sequence are mutation site.

Primer Name	Primer Sequence 5′–3′	Application
Sox5 F	TGGCAATGGGATCAGGGAAC	Real time PCR (Polymerase Chain Reaction)
Sox5 R	ATCATACCCATGAGCTGCCG	Real time PCR
Sox5 3′ UTR F	CCGCTCGAGGCTGACAAATAGACCTCAGCC	Luciferase reporter-wt
Sox5 3′ UTR R	ACGCGTCGACTGTTGGCACTGTGTACCTGA	Luciferase reporter-wt
Sox5-3′ UTR-mut F	GAAGAAGAGATCGAAATTTCGTATGGGTTAAACAGTGCC	Luciferase reporter-mut
Sox5-3′ UTR-mut R	GGCACTGTTTAACCCATACGAAATTTCGATCTCTTCTTC	Luciferase reporter-mut
miR-21a F	ACACTCCAGCTGGGTAGCTTATCAGACTGAT	Real time PCR
Universal Primer	TGGTGTCGTGGAGTCG	Real time PCR
U 6 F	CTCGCTTCGGCAGCACA	Real time PCR
U 6 R	AACGCTTCACGAATTTGCGT	Real time PCR
miR-21a R	CTCAACTGGTGTCGTGGAGTCGGCAATTCAGTTGAGGTCAACAT	Real time PCR
MITF F	CAGCAACGAGCTAAGGACC	Real time PCR
MITF R	GGATGGGATAAGGGAAAGT	Real time PCR
18S-F	GAAGGGCACCACCAGGAGT	Real time PCR
18S-R	CAGACAAATCACTCCACCAA	Real time PCR
TYR-F	ACTTACTCAGCCCAGCATCC	Real time PCR
TYR-R	AGTGGTCCCTCAGGTGTTCC	Real time PCR
